# Mechanical ventilation in snake envenomation of dogs and cats

**DOI:** 10.3389/fvets.2023.1071257

**Published:** 2023-03-29

**Authors:** Cameron A. D. Morris, Rebekah E. Donaldson

**Affiliations:** Critical Care Department, Queensland Veterinary Specialists, Brisbane, QLD, Australia

**Keywords:** *Elapidae*, positive pressure ventilation, neuromuscular disease, neurotoxicity, *Micrurus fulvius*, *Pseudonaja*, *Notechis*, *Pseudechis*

## Abstract

Envenomation by snakes in *Elapidae* and *Viperidae* families have been associated with respiratory failure in dogs and cats. Mechanical ventilation may be required for hypoventilation due to neuromuscular paralysis or hypoxemia due to pulmonary hemorrhage or aspiration pneumonia. Median incidence of dogs and cats with snake envenomation that require mechanical ventilation is 13% (0.06–40%). Standard treatment of snake envenomation in dogs and cats includes prompt administration of appropriate antivenom and management of envenomation complications such as coagulopathy, rhabdomyolysis and acute kidney injury. When mechanical ventilation is required, overall prognosis is good with appropriate treatment. Standard anesthetic protocols and mechanical ventilator settings are generally appropriate, with lung protective ventilation strategies typically reserved for patients with pulmonary disease. Median survival to discharge for cats and dogs with elapid envenomation is 72% (76–84%) with 33 h (19.5–58 h) median duration of mechanical ventilation and 140 h (84–196 h) median hospitalization. This article reviews indications for mechanical ventilation in cats and dogs with snake envenomation, and discusses ventilator settings, anesthetic and nursing considerations, complications and outcomes specific to this disease.

## 1. Introduction

A variety of venomous snake species exist globally, with envenomation leading to neuromuscular and hemostatic disorders (1). The mainstay of therapy in these patients includes antivenom (appropriate type and time frame) and supportive treatment including intravenous fluid therapy, analgesia, nutrition, and management of complications.

Ventilatory support is required for those with severe neuromuscular disease resulting in respiratory compromise. There is a paucity of evidence in the veterinary literature on the ideal use of mechanical ventilation (MV) for cats and dogs undergoing treatment for snake envenomation. The median incidence of dogs and cats with snake envenomation that require MV is 13% (0.06–40%) (2–10).

This review aims to discuss existing literature regarding mechanical ventilation in envenomated dogs and cats, including patient management and prognosis, and identify areas for future research.

## 2. Pathophysiology

### 2.1. Snake species

While envenomation by numerous snake species has been associated with respiratory failure in cats and dogs, only snakes in the *Elapidae* and *Viperidae* families have caused documented respiratory paralysis (11).

Elapid snakes, belonging to the *Elapidae* family, are characterized by a pair of rostrally positioned fangs (1, 12). Venom constituents and effects vary between species (12). Neurotoxin induced respiratory paralysis results in hypoventilation requiring mechanical ventilation in severe cases. Envenomation can pose a significant threat to life should treatment be delayed (13).

Elapids are distributed globally but the majority of species are located within North America, South-eastern Asia and Australia. Australian elapids have been reported as some of the most venomous species on earth (14). Those of significance include the Brown snakes (*Pseudonaja* spp.), Tiger snakes (*Notechis* spp., *Austrelaps* spp.), Black snakes (*Pseudechis* spp.), Taipans (*Oxyuranus* spp.), and the death adder (*Acanthophis* spp.) (15). Mechanical ventilation was most commonly required for hypoventilation in Australian dogs and cats envenomated by brown snake (19%, 14/59), tiger snake (11%, 5/46), and black snake (11%, 1/9) immunotypes in a retrospective multicenter study (7). Hypoventilation requiring MV has been documented in one case of *Pseudechis prophyriacus* envenomation (16). Hypoxemia secondary to pulmonary hemorrhage is a more common indication for MV in envenomation by this species (4, 7).

Kraits (*Bungarus* spp.), Cobras (*Naja* spp.), and the King Cobra (*Ophiophagus hannah*) are *Elapidae* that inhabit Asia. No studies in the veterinary literature describe the use of MV for envenomation by these species. One case report exists of two children suffering *Bungarus caeruleus* and *Naja naja* envenomation, requiring 10 and 11 days of invasive MV, respectively (17). Another human study reported 51% (17/33) of patients required MV for *B. caeruleus* envenomation, with all patients alive at the 9 month follow up after a median of 96 h of ventilation (18).

Eastern coral snakes (*Micrurus fulvius*) are *Elapidae* found in North America. Envenomation by *Micrurus* sp. has been documented to cause respiratory paralysis in cats and dogs necessitating mechanical ventilation, with a median duration of MV 58 h (25–84 h), median duration of hospitalization of 8.2 days (6–11 days) and survival rate of 87.5% (7/8) (19).

Within North America, envenomation is more commonly reported from members of the *Viperidae* family—Rattlesnakes (*Crotalus* spp.), and the Copperheads (*Agkistrodon* spp.) (20). Rattlesnakes account for most mortalities in this area but the severity of neurological signs varies between species (6). Positive pressure ventilation (PPV) was indicated or provided in between 1 and 11.8% (1/82 and 4/34) rattlesnake envenomation's in reviews of dogs with *Crotalus* spp. envenomation (6, 21). MV for *Agkistrodon contortrix* envenomation has not been documented in dogs or cats (22).

### 2.2. Toxin classification

There are several recognized toxin types, which can be divided into neurotoxins, coagulants (both pro- and anti-coagulant), myotoxins, hemolysins, and cytotoxins (23, 24). As neurotoxins are the primary cause for respiratory paralysis, they shall be the focus of this article. Additional consideration should, however, be given to coagulants and myotoxins for their potential contributory role in the need for MV, as well as their influence in disease progression and outcome.

Two main types of neurotoxins inhibit synaptic transmission at the neuromuscular junction (NMJ), phospholipase A2 (PLA2) enzymes and three-finger alpha-neurotoxins. PLA2 enzymes irreversibly bind and hydrolyze the membrane phospholipids at the motor nerve terminal. This results in a calcium influx and subsequent release and depletion of acetylcholine, leading to degeneration of the nerve terminal (25). These include ammodytoxin A (*Vipera ammodytes*), β-bungarotoxin (*Bungarus multicinctus*), taipoxin (*Oxyuranus scutellatus*), and textilotoxin (*Pseudonaja textilis*) (26). Eastern coral snake venom contains PLA2 enzymes and a type of alpha-neurotoxin, a post-synaptic acetylcholine receptor antagonist, resulting in generalized muscle weakness and respiratory paralysis (2, 19). Alpha-neurotoxins, such as α-Bungarotoxin, bind and inhibit post-synaptic acetylcholine receptors at the NMJ (25, 27).

PLA2 enzymes also play a role in myotoxicity, causing either local swelling at the bite site, rhabdomyolysis, or both (28). Enzymes such as notexin, found in *Notechis scutatus* venom, cause extensive skeletal muscle damage, including muscles responsible for respiration (26). Regeneration of peripheral nerves begins 3–4 days following envenomation, with almost complete resolution of damage by 10–14 days (25). Rhabdomyolysis has been associated with the development of acute kidney injury (AKI) which may alter the patient's management and prognosis (25, 27, 29).

Coagulation disorders may cause respiratory failure by pulmonary thromboembolism or pulmonary hemorrhage (5). Elapids with procoagulant toxin resulting in consumption of clotting factors (known as venom-induced consumptive coagulopathy) include *Pseudonaja* spp., *N. scutatus*, and *Oxyuranus* spp. (30). Anticoagulant toxin present in *Pseudechis* spp., *O. hannah*, and *Naja* spp. inhibits factors X, II and platelet function (30–32).

### 2.3. Clinical signs

Interference at the NMJ leads to progressive lower motor neuron signs. Clinical signs can range from generalized paresis to paralysis and death from respiratory failure (19). Localized swelling of the bite site can occur with certain snake species, and hemorrhage may be witnessed if the venom contains hemolytic toxins (16, 33). In Australian elapids, bite sites are often not found due to small sized fangs and minimal localized swelling in combination with thick hair coats of cats and dogs (34). Other common clinical signs include vomiting, hypersalivation, diarrhea, tachypnea, collapse, mydriasis, ataxia, and pigmenturia (4, 19, 33, 35).

Clinical signs typically are acute in onset (often within 1 h) but can be biphasic or delayed. Retrospective studies report the majority of envenomated patients are presented for veterinary assessment within 6 h (dogs) to 3 days (cats) (33, 36). The incidence of dogs and cats that are asymptomatic on presentation is unknown. Humans asymptomatic for known snakebites are monitored for 12–24 h in hospital, should testing be inconclusive (37, 38). One study reported development of clinical signs up to 14 h after *Micrurus fulvius* envenomation and a previous controlled experimental study showed development of paralysis within 24 h following subcutaneous administration of *Pseudonaja* sp. venom in cats (35, 39).

## 3. Patient management

### 3.1. Antivenom

Antivenom is a crucial part of treatment for any patient with clinical evidence of snake envenomation. Antivenom is produced by injecting venom or components into a host (most commonly horses) and extracting the newly formed IgG antibodies from the plasma (40, 41). IgG antibodies bind to the active unbound venom antigen, forming masses of immune complexes that are readily phagocytized, inhibiting attachment to receptor sites (42). Commercially available antivenom products are summarized in Table 1.

**Table 1:** Commercially available antivenom therapies.

**Antivenom**	**Concentration**	**Indicated species**	**Recommended vials**
Padula Serums Tiger-Brown Bivalent^a^	4,000 units Tiger snake, 4,000 units Brown snake.	*Notechis scutatus Pseudonaja textilis Pseudechis* spp.	2 (5)
Padula Serums Brown Snake^a^	2,000 units	*Pseudonaja* spp.	1
AVSL Multi Brown Snake^b^	1,600 units	*Pseudonaja* spp.	1–2
Summerland Serums Tiger/Multi-Brown^c^	4,050 units Tiger snake, 4,050 units Brown snake.	*Pseudonaja* spp. *Notechis scutatus Pseudechis* spp.	1
Summerland Serums Multi-Brown^c^	2,050 units	*Pseudonaja* spp.	1
Seqirus Tiger Snake^d^	3,000 units	*Notechis scutatus Pseudechis* spp.	1
Coralmyn^e^	5 mg	*Micrurus* spp.	1–2 (2)
Crotalidae Polyvalent Immune Fab (CroFab)^f^	-	*Crotalus* spp. *Agkistrodon* spp.	1–2 (6)
Antivenin Crotalidae Polyvalent (ACP)^g^	-	*Crotalus* spp. *Agkistrodon* spp.	1–2 (21)
VenomVet Crotalidae Polyvalent^h^	-	*Crotalus* spp. *Agkistrodon* spp.	1–2 (41)

a Snake Antivenom, Padula Serums, Bairnsdale, Australia.

b Multi Brown Snake Antivenom, Australian Veterinary Serum Laboratories (AVSL), Lismore, Australia.

c Tiger Multi-Brown Antivenom, Summerland Serums, Alstonville, Australia.

d Tiger Snake Antivenom, Commonwealth Serum Laboratories Seqirus, Parkville, Victoria, Australia.

e Coralmyn, Instituto Bioclon, Mexico City, Mexico.

f CroFab, BTG Pharmaceuticals, Conshohocken, PA, USA.

g Antivenin Crotalidae Polyvalent, Boehringer Ingelheim Inc, Duluth, GA, USA.

h VenomVet, MT Venom LLC, CA, USA.

Antivenom varies by geographical location. Correct identification of individual snake species can be difficult, yet fortunately is less crucial with the development of polyvalent antivenoms. In Australia, snake venom detection kits are utilized to aid in selection of the most appropriate antivenom therapy.^1^ Specificity of this ELISA (Enzyme-linked Immunosorbent Assay) has been reported as 100%, however false negatives from acute envenomation, absence of circulating venom and the “hook effect,” whereby binding of antibodies in the presence of a high venom concentration inhibits sandwich formation, should be considered (33, 43, 44). This immunoassay can be performed patient side with a rapid result (5).

The ideal dose of antivenom is unknown. Research into *Pseudonaja textilis* envenomation identified neutralization of venom levels following administration of 4,000 units each of an experimental IgG tiger—brown snake antivenom (5). A previous study used between 3,000 and 6,000 units for *N. scutatis* envenomation in cats and dogs (13). A study of cats undergoing MV for elapid envenomation in Australia reported ~42% (5/12) of cats received one vial, 42% (5/12) received two vials and 16% (2/12) of cats received 3 vials of antivenom, containing 3,000 units of *N. scutatis* antivenom with 1,000 units of *P. textilis* antivenom or 3,000 units of *N. scutatis* with 4,000 units of *P. textilis* antivenom (45). This study did not evaluate outcomes between the two different antivenom treatment groups, however 11 of the 12 patients survived to discharge. Another study investigating elapid envenomation in Melbourne, Australia administered a median of 8,100 units of tiger/brown polyvalent antivenom to dogs and a median of 4,050 units to cats (9). Most cats and dogs with *Micrurus sp*. snakebite receive 5–10 mg of IgG equine-derived antivenom (2, 19). A mean of five vials of a polyvalent antivenom was reported in one canine study in Sri Lanka, with each vial capable of neutralizing 6 mg *N. naja*, 4.5 mg *B. caeruleus*, 6 mg *D. russelii*, and 4.5 mg *E. carinatus* venom (46). In humans, current guidelines support giving one vial (1,000 units) in both children and adults for most elapid species (37).

There is no evidence to suggest additional vials are of benefit to justify the increased risk associated with repeated antivenom administration (37). For all other elapid species, following human guidelines, one vial of appropriate antivenom should be provided with additional vials given if clinical deterioration is observed or if a repeated snake venom detection kit is positive (9, 37). Additional antivenom may be required in the event of multiple bites, however the number of bites may be irrelevant as dry bites can occur without release of venom (47). A maximum number of antivenom vials cannot be suggested based on available evidence, with further investigation into antivenom dosages required.

In a study of dogs and cats envenomated by *P. porphyriacus*, ventilated patients did not require more antivenom compared to non-ventilated patients (4). There was no significant difference between number of vials of antivenom and length of hospitalization or survival in another study of dogs and cats undergoing MV for rattlesnake envenomation (6). Antivenom is most effective when administered as soon as possible to reduce the need for mechanical ventilation given its inability to neutralize bound pre-synaptic toxin (48). Post-synaptic neurotoxins will be neutralized following antivenom administration, therefore a cat or dog presenting with snake envenomation in which mechanical ventilation is indicated will not be expected to immediately improve with antivenom administration and mechanical ventilation should proceed (45).

Antivenom should be diluted prior to administration (49). The rate of administration in humans is not associated with an increase in adverse effects (50). With any blood product, initial administration should be commenced slowly with the rate titrated, however in the face of lethal envenomation, antivenom has been reported to be given in as little as 5 min (10). Multiple studies report administration either rapidly (administered as a bolus) or over a 20 min period (5, 6, 10, 19). Rapid administration is anecdotally tolerated at the authors' institution, but no veterinary literature exists evaluating the incidence of transfusion reactions. There is no evidence to support premedication of patients with antihistamines or glucocorticoids prior to administration of antivenom (3, 51, 52).

### 3.2. Indications for mechanical ventilation

There are currently four recognized indications for mechanical ventilation: (1) severe hypoxemia defined as partial pressure arterial oxygen (PaO_2_) <60 mmHg despite oxygen therapy (fraction of inspired oxygen (FiO_2_) >50%); (2) severe hypoventilation defined as partial pressure arterial carbon dioxide (PaCO_2_) >60 mmHg; (3) unsustainable respiratory effort, and (4) hemodynamic compromise unresponsive to traditional therapy (53–55).

Hypoventilation and respiratory fatigue are the more common indications for MV in the envenomated patient. Hypoxemia is less frequently reported but can occur due to severe pulmonary hemorrhage or aspiration pneumonia (10, 45, 56–58).

## 4. Ventilator settings

The aim of MV in envenomated cats and dogs is to provide ventilatory support whilst the patient processes the bound neurotoxins, targeting appropriate oxygen and carbon dioxide levels using minimally aggressive ventilator settings (53).

### 4.1. Mode of ventilation

Of the studies published describing MV in elapid envenomation, few outline the ventilator settings used. Compared to other patients with lower motor neuron diseases requiring MV, cats and dogs with elapid envenomation generally lack primary pulmonary disease and require short ventilation periods and minimally aggressive settings (45, 58).

One retrospective study of eight cats and dogs with *M. fulvius* envenomation performed volume-controlled positive pressure ventilation using synchronized intermittent mandatory ventilation (SIMV), with initial pressure support of 8 cm H_2_O and positive end expiratory pressure (PEEP) 3 cm H_2_O. This study reported a median duration of reliance on mandatory breaths of 15 h (8–24 h), with a median duration of ventilation of 58 h (25–84 h) and 7/8 patients were successfully weaned (19). In a study of 12 cats, the authors followed a pre-established protocol using volume-controlled positive pressure ventilation with a respiratory rate of 20 breaths/min, tidal volume of 10 ml/kg, PEEP of 3–5 cm H_2_O and a spontaneous breathing trial performed every 12 h (45). Eleven of the 12 cats were successfully weaned, with a median duration on MV of 19.5 h (7–37 h). In a case report, one dog with *P. porphyriacus* envenomation was managed using pressure-controlled ventilation and was weaned after 18 h, however the breath pattern was not reported (16).

Invasive ventilation facilitates airway protection so is preferred to non-invasive ventilation in cats and dogs with generalized neuromuscular disease. Dynamic positive airway pressure systems such as high flow nasal oxygen therapy are currently not considered appropriate if the patient's primary cause of respiratory failure is hypoventilation (59).

No veterinary literature compares pressure vs. volume controlled mandatory modes of ventilation. It has been suggested that pressure-controlled ventilation may reduce the work of breathing and therefore improve patient comfort and patient-ventilator synchrony (60). Mode of MV is uncommonly documented in the human literature with specific reference to snake envenomation. Successful ventilation with survival to discharge is documented in case reports using SIMV and mandatory pressure-controlled ventilation for *Bungarus* sp. envenomation (61, 62). Volume-controlled mandatory ventilation was compared to adaptive support ventilation in a prospective randomized trial of 48 adults with neuroparalytic snake envenomation; mode did not appear to significantly affect outcome with respect to time to weaning, incidence of VAP or length of hospital stay (63). Median duration of MV was 17 h in a prospective study of adult envenomated humans (64). Of these, 60% (8/14) patients required MV for <24 h and 13/14 (93%) survived to discharge (64). Mode of MV was not specified in this study. Further research is required in both human and veterinary medicine. At the authors' institution, invasive ventilation is most commonly performed in mandatory pressure-controlled mode with anecdotal success.

### 4.2. Oxygen concentration

Patients should be initially commenced on FiO_2_ 100% at the start of MV. Oxygen concentration is titrated down incrementally if adequate arterial oxygenation can be maintained, as assessed by pulse oximetry or preferably arterial blood gas analysis (65). To reduce the risk of oxygen toxicity and minimize absorption atelectasis, this process should commence as soon as the patient is stable on the ventilator. Targeting the lowest possible FiO_2_ concentration to maintain an SpO_2_ or SaO_2_ at >95% (66).

### 4.3. Pressure, volume, and frequency

Lung protective ventilation involving reduced inspiratory pressures (<30 cmH_2_O), lower tidal volumes (6–8 ml/kg), higher PEEP and presence of permissive hypercapnia have been associated with reduced mortality in people with acute lung injury and acute respiratory distress syndrome (ARDS) (67, 68). This approach should be considered for envenomated cats and dogs with pulmonary disease such as hemorrhage or pneumonia (69). For cats and dogs without pulmonary disease, initial recommended setting includes tidal volume 8–12 ml/kg, respiratory rate 10–20 breaths per minute, inspiratory pressures of 10–20 cm H_2_O and inspiratory to expiratory ratio 1:2 s. Low levels of PEEP (3–5 cm H_2_O) are described to avoid atelectasis (70). In all cats and dogs receiving MV, ventilator settings should be adjusted to the least aggressive settings required to achieve normotension and normoxemia (69).

## 5. Anesthetic considerations

Total intravenous anesthesia is typically used to facilitate MV. Injectable anesthetics and sedatives should be selected based on the patient's underlying comorbidities, hemodynamic stability and ventilator tolerance (71). Reversible and short-acting injectable agents are preferred for patient safety.

To the author's knowledge, no specific anesthetic protocol exists for veterinary patients undergoing ventilation for snake envenomation. However, in the literature available, the more commonly used protocols for envenomated cats and dogs undergoing MV include a combination of a mu-agonist opioid, a benzodiazepine and propofol titrated by constant rate infusion (CRI) (8, 16, 19, 45). This combination is effective in the envenomated cat or dog given the short duration of action and minimal effect on neuromuscular transmission (72). Multi-drug protocols allow a reduction in the side effect profile of each agent and may reduce recovery time (73).

Propofol may be considered at 4–6 mg/kg IV for induction followed by 0.1–0.6 mg/kg/min CRI given its fast onset and short duration (74). Lower doses may be sufficient given the severity of paralysis experienced by patients with elapid envenomation (5, 75). Recognized adverse effects include hypotension and Heinz body anemia in cats receiving multi-dosing or CRIs (76, 77).

Pure-mu opioid agonists (e.g., fentanyl) provide analgesia and sedation with the benefit of being short acting and reversible, which may facilitate spontaneous breathing trials during weaning. Dose dependent respiratory and cardiovascular depression can occur. Reported fentanyl doses are 2–5 mcg/kg IV bolus prior to 1–10 mcg/kg/h CRI (73, 74). Butorphanol, a pure mu-opioid antagonist and kappa agonist, can also be used to mechanically ventilate dogs and cats with neuromuscular disease (5, 19, 74). From an analgesic perspective, use of pure mu-agonists is recommended in cats and dogs experiencing rhabdomyolysis secondary to envenomation (33).

Benzodiazepines (e.g., midazolam and diazepam) provide sedation and muscle relaxation and have been shown to be safe and effective in combination with fentanyl and propofol in dogs (78). Midazolam has a lower thrombophlebitis risk than diazepam (71, 78). Respiratory depression can occur and hyperexcitability has been reported in cats (74). Benzodiazepine withdrawal leading to seizure activity has been reported in humans and more recently in young dogs following MV at doses of 0.2–1.5 mg/kg/h (79, 80). These dogs developed generalized seizure activity within 36 h of weaning from MV, and were treated with tapering doses of midazolam, as well as levetiracetam in one dog. No ongoing abnormal neurological activity was reported (79). Recommended dose ranges from 0.1 to 0.5 mg/kg/h CRI (19, 73).

Alpha-2 adrenergic agonists (e.g., medetomidine and dexmedetomidine) are sedating with minimal respiratory depression (81). Alpha-2 agonists can reduce anesthetic drug requirements by up to 80% in cats and dogs (81, 82). Adverse effects in cats and dogs include cardiovascular depression and atrioventricular block, as well as vomiting in cats (74). Dexmedetomidine CRI was included in the anesthetic protocol for a puppy that was successfully ventilated for respiratory paralysis due to polyradiculoneuritis and has been reported as an appropriate agent for dogs and cats mechanically ventilated with *M. fulvius* envenomation (19, 75). This paper did not specify if dogs only or both dogs and cats received dexmedetomidine as part of their TIVA protocol. Recommended dose rates as an adjunct for MV include 0.5–3 mcg/kg/h CRI (83).

As almost all cats and dogs requiring MV due to snake envenomation will have neuromuscular paresis or paralysis, the administration of neuromuscular blocking agents (NMBA) is not necessary (16, 45, 84). Rocuronium use to facilitate intubation in humans with *Naja kaouthia* envenomation was described in a case series, which more likely reflects species differences in airway management rather than ongoing concern for patient-ventilator dyssynchrony (85). Another clinical trial evaluating 14 envenomated humans did not use NMBA during MV (64).

All patients undergoing MV should have continuous monitoring, including invasive or non-invasive blood pressure monitoring, arterial or venous blood gas sampling, electrocardiography, capnography and pulse oximetry (53).

No standardized anesthesia protocol exists for envenomated humans undergoing MV. A variety of drug protocols have been described, commonly including midazolam and fentanyl (63, 85). Additional therapies have been described in humans to improve neuromuscular function. Cholinesterase inhibitors (such as neostigmine, edrophonium) have been used in numerous cases of envenomated humans. Their use may reduce the effect of post-synaptic toxins by increasing acetylcholine concentrations but would have no effect on post-synaptic toxins or delayed presentations (64, 85–87). Two dogs treated with neostigmine in a case series of dogs with neuroparalytic snake envenomation showed no improvement or mild neurological improvement insufficient to change clinical treatment (88). Calcium gluconate has been used for its role as a neurotransmitter and was described in three cases of humans with *Bungarus caeruleus* envenomation to anecdotal success (17, 89). The patients described received standard care including antivenom and cholinesterase inhibitors and treatment response was not objectively quantified. The clinical utility of this therapy in veterinary patients is unclear.

## 6. Ancillary considerations

### 6.1. Nutrition

Provision of a balanced diet at an appropriate proportion of RER based on individual nutritional assessment is beneficial for patients undergoing MV and should be considered for snake envenomated cats and dogs after 72 h of hospitalization (90).

There are no published guidelines on re-alimentation in mechanically ventilated veterinary patients (91–96), and the frequency of nutrition provision in mechanically ventilated cats and dogs remains poor (90). Veterinary patients without appropriate nutritional support are at risk of starvation. Starvation may prolong mechanical ventilation in the envenomated patient due to impaired respiratory muscle function from respiratory muscle catabolism, reduced surfactant production, decreased immune response and hypoalbuminemia that may exacerbate pulmonary edema (55, 91). Excessive caloric intake increases carbon dioxide production which may affect MV settings (91).

Enteral nutrition (EN) is preferred as it is associated with decreased mortality and duration of hospitalization and more ventilator-free days in humans (92, 97). Physiological benefits include maintenance of gastrointestinal integrity as well as modulation of stress and the immune response (92–95, 98). Nasogastric/nasoesophageal feeding tubes are appropriate as envenomated patients generally have a short disease course without ongoing gastrointestinal dysfunction.

Trophic feeding is preferred to total caloric provision, with one study in humans reporting a reduced incidence of gastrointestinal intolerance to EN but no overall difference in ventilator free days or mortality (92, 96). Patients who are likely to tolerate EN should be given a balanced, digestible commercially available veterinary diet (99). One study in hospitalized dogs requiring nasogastric or nasoesophageal feeding described the use of veterinary (Clinicare^2^ and Clinicare RF^3^) and human (Ensure^4^) liquid enteral diets with no significant difference in outcome (90, 92, 100, 101). Other diets have been described in two dogs receiving EN at 25% RER (Royal Canin Convalescence Support Instant Diet^5^ and Enteral Care KC^6^) (90). At the authors' institution, patients undergoing MV are initially fed at 25% RER and increased in 25% increments according to the patients' tolerance to feeding.

Parenteral nutrition (PN) can be provided as adjunctive nutrition for patients that may not tolerate EN (such as those with severe ileus) or require supplementation to an EN feeding plan (100). Administration requires strict aseptic technique, intensive monitoring, and a specific catheter to reduce the risk of complications (100, 101). PN has not been evaluated in mechanically ventilated veterinary patients but based on human literature, it should not be used before or in preference to EN (90, 92, 93).

### 6.2. Gastrointestinal considerations

Gastrointestinal dysmotility is a reported side effect of snake envenomation, which may be worsened by shock or mechanical ventilation (102). Megaesophagus has been documented in a case report of four dogs with tiger snake (*Notechis scutatus)* envenomation (56).

MV can cause relaxation of the lower esophageal sphincter and the development of gastroesophageal reflux as a consequence of intrathoracic pressure changes and anesthetic agents used (103). Prokinetic therapy is indicated if gastric ileus is identified ultrasonographically or if gastric residual volumes exceed 10 ml/kg (93, 103). Metoclopramide 2 mg/kg/d CRI is an appropriate first line prokinetic in dogs (103). Proton pump inhibitors are indicated only when there is evidence of gastrointestinal ulceration or esophagitis to reduce the risk of VAP development, given gastrointestinal signs are generally brief and resolve with antivenom and anti-emetics (33, 104).

### 6.3. Nursing care

Mechanically ventilated cats and dogs require continuous care and are at risk of complications including ocular and oral ulceration, decubital ulcers and aspiration of gastric fluid (71). Suboptimal patient care is associated with increased mortality in humans (105, 106).

Envenomated cats and dogs may be at greater risk of ocular ulceration due to tear desiccation, the inability to blink and reduced tear production in the critically ill (107). One study describing MV in Australia reported corneal ulceration in 58% (7/12) of envenomated cats, and a study of *M. fulvius* envenomation in dogs and a cat reported corneal ulceration in 25% (2/8) (19, 45). Other studies in dogs and cats describing MV for mixed causes report the incidence of corneal ulceration between 5 and 10% (69, 108). Regular sterile saline lavage, daily fluorescein stains, and administration of lubricating ointment every 2 h is recommended to reduce and prevent the risk of exposure keratitis (71). Polyethylene eye covers are utilized in human ICU settings, with one veterinary study reporting use of lenses in envenomated cats and dogs undergoing MV (5, 109). Further investigation of protective ocular equipment and their use in veterinary patients is required (110, 111). Development of blepharospasm, conjunctival hyperemia, mucoid discharge and episcleral congestion should prompt clinicians to investigate and escalate ocular care. Superficial corneal ulcers require treatment with a broad-spectrum antimicrobial ointment with close monitoring for progression.

Frequent oral care and airway management can reduce the likelihood of oral ulceration, lingual oedema, aspiration of gastric contents and bacterial colonization to lower risks of ventilator associated pneumonia (VAP). Complete oral care protocols are described elsewhere (71), but should be performed every 4 h, incorporating appropriate hand hygiene and use of sterile gloves, cleaning of the oral cavity and attachments with a 0.05% chlorhexidine solution and regular suctioning. Frequent deflation and repositioning of the endotracheal tube cuff has been recommended after suctioning to reduce the risk of pressure necrosis in cats and dogs, however is associated with increased risk of VAP in humans (112, 113). Ensuring cuff pressure is maintained at 25 cm H_2_O through the use of a commercial endotracheal tube cuff inflation device is recommended to reduce the development of VAP and pressure necrosis (71, 112, 114). If a monitoring device is unavailable, the cuff should be deflated and repositioned to reduce the risk of tracheal necrosis (114).

Indwelling urinary catheterization allows close monitoring of urine production and increases patient comfort, reducing the potential for patient-ventilator asynchrony. Aseptic technique and a closed collection system facilitates gross assessment of urine, sampling for urinalysis and accurate urine output measurement. Appropriate catheter care is required to minimize urinary tract infection risk. Acute kidney injury secondary to envenomation is documented in several studies, thought to be a consequence of venom-induced hemolysis or rhabdomyolysis leading to pigmenturia (8, 19, 115, 116).

Prolonged patient recumbency can result in decubital ulcers, generalized muscle weakness and edema development, with ulcer severity associated with increased mortality in humans (114, 117). Patients undergoing long term ventilation (>48 h) are at a higher risk. Passive range of motion exercises and regular recumbency changes are recommended every 4 h, with padded bedding provided to maximize patient comfort.

### 6.4. Fluid therapy

Veterinary literature reports the use of isotonic crystalloids as fluid therapy for snake envenomated cats and dogs (8, 16, 19, 118), although many did not specify the type of fluid and the optimal rate is unclear. Intravenous fluid therapy should aim for neutral fluid balance, providing maintenance fluid requirements in combination with correcting dehydration, shock or ongoing losses where required. Human literature suggests a positive fluid balance significantly increases the risk of ventilator-associated adverse events (119).

### 6.5. Blood products

Red blood cell containing products such as packed red blood cells (pRBC) or fresh whole blood (FWB) may be required in cases of severe hemolysis or hemorrhage. Packed red blood cell therapy was administered in 14/88 patients with *P. porphyriacus* envenomation that developed severe anemia with 57% survival (8/14) (4).

Fresh frozen plasma (FFP) therapy is controversial in snake envenomation. Snake envenomation causes hemorrhage due to venom induced consumptive coagulopathy (VICC) (120). Procoagulant toxins causing VICC can be neutralized by snake antivenom in *in-vitro* models (5, 10). However, VICC still occurs despite prompt antivenom treatment due to the rapid effects of these toxins. Human literature recommends prioritization of antivenom administration, with administration of FFP only in instances of ongoing bleeding (121). A small randomized clinical trial of humans administered FFP within 4 h of antivenom treatment failed to demonstrate improvement in clinical outcomes or time to resolution of clinical coagulopathy, although clotting factor dysfunction resolved more rapidly (121).

Veterinary literature has documented the administration of FFP for severe hemorrhage following antivenom administration. In 10 dogs with severe pulmonary hemorrhage secondary to *P. textilis* envenomation, 70% (7/10) were administered FFP. Of these, 43% (3/7) survived to discharge (10).

## 7. Complications

Mechanical ventilation risks complications that should be considered and discussed with clients prior to commencement. Commonly reported complications include VAP, pneumothorax, ventilator-induced lung injury (VILI) and cardiovascular compromise (53, 69). The potential for development of aspiration pneumonia is an additional consideration, however, this was not a complication identified in some of the larger study populations of cats and dogs ventilated for elapid envenomation (45, 58). Aspiration pneumonia is reported as a secondary complication of envenomated patients with transient megaesophagus (56, 57). VAP is defined as pneumonia that arises 48 h after endotracheal intubation (122). Prolonged duration of MV has been associated with a higher likelihood of VAP, and reduced survival (45, 108, 122).

None of the 12 cats in one study ventilated for elapid envenomation developed VAP or a pneumothorax, likely due to the short duration of MV and minimally aggressive ventilator settings (45). In another study of seven dogs and one cat managed for eastern coral snake envenomation, ventilator associated complications were noted in all patients with pneumonia in 62% (5/8) (19). Pneumonia was diagnosed radiographically in all patients, with positive bacterial cultures obtained in 4/8 patients. It is unclear if these patients had VAP or pre-existing pneumonia as thoracic radiographs were not obtained when MV was initiated, and airway sampling was not performed in all animals (19).

Pneumothorax is a rarely reported complication in the veterinary literature (2, 4, 5, 7, 8, 16, 19, 45). One study of 302 cats and dogs undergoing MV for all causes reported a lower prevalence of pneumothorax compared to previous literature (58). It did not, however, specify the number of pneumothoraces identified or whether any were present in the snake envenomation subgroup (58). The low incidence of pneumothorax in this cohort is likely a reflection of the lack of pulmonary disease in this patient group as well as the conservative ventilator settings required to effectively ventilate patients experiencing respiratory failure due to hypoventilation. Thoracostomy tubes are required should a pneumothorax develop during the ventilation period (70).

ARDS is an uncommonly described complication of envenomated cats and dogs undergoing MV. Three studies reported a single case each (4, 19, 123). The low incidence of ARDS is likely because these animals generally lack lung pathology and are ventilated for relatively short durations.

AKI is defined as an increase in creatinine by 26.5 umol/L (0.3 mg/dL) from baseline (124). In this patient population, AKI can occur secondary to rhabdomyolysis, hemolysis, reduced renal perfusion consequent to increased intrathoracic pressures from MV or hemorrhagic shock (4, 19, 125, 126). AKI was reported in 5.7% (5/88) of dogs and cats in one study assessing *P. porphyriacus* envenomation, with 80% (4/5) animals being euthanized due to development of anuria or overall deterioration (4). Two of eight (25%) cats and dogs with *M. fulvius* envenomation in another study were found to develop an AKI, of which only one survived (19).

AKI is reported in envenomated dogs independent of MV, with a retrospective study identifying AKI in 29% (16/56) of dogs with pit viper envenomation within 48 h of presentation (127). Dogs with an AKI had an increased risk of mortality associated with the severity of shock and increased doses of antivenom (127). AKI is reported in 30–57.8% of human patients suffering from neurotoxic elapid and vasculotoxic viper envenomation (128, 129). Prolonged bite-to-hospitalization period is associated with increased risk of AKI and has been associated with worse outcomes (including mortality, need for MV, and renal replacement therapy) (128, 129). Management of AKI in these patients should be focused on maintaining an appropriate fluid balance and increasing renal perfusion by limiting intrathoracic pressures (130).

Coagulopathy is a feature of envenomation by specific elapid species (33). Hemotoxins may lead to pulmonary hemorrhage and complicate patient recovery as MV is then required to address both hypoxemia and hypoventilation. Venom-induced consumptive coagulopathy was the most common reported disorder (73%, 611/835) for humans in the most recent Australian Snakebite Project (131).

No significant complications including AKI, cardiac arrhythmias, SIRS, MODS or hemorrhage were reported in any patient receiving antivenom within 6 h of being bitten by *P. porphyriacus* (4). Early administration of antivenom in envenomated patients has been shown to reduce the incidence of complications (132).

## 8. Weaning from mechanical ventilation

Ventilated cats and dogs must meet specific criteria prior to attempting weaning from MV (133). Long term ventilation leads to respiratory muscle weakness which increases with the duration of time on the ventilator (133). Those with improvement in their primary disease, a PaO_2_:FiO_2_ ratio > 150–200 on an FiO_2_ < 0.5, minimal PEEP requirements, hemodynamic stability and an appropriate respiratory drive can be considered candidates for a weaning trial (133).

The most commonly used weaning modes are synchronized intermittent mandatory ventilation (SIMV), or spontaneous breathing trials (SBT) using either pressure support ventilation (PSV) or disconnection from the machine. SBT's are commonly performed in human medicine as they allow the patient to increase respiratory muscle strength over short, regular intervals (134). One case report of a dog ventilated for *P. porphyriacus* envenomation was successfully weaned with a SBT and extubated after 4 h, but it did not specify how this was performed (16). None of the larger retrospective ventilation studies described the method of weaning used or duration of the process (19, 45, 58). As nerve terminal regeneration is reported to occur from 3 to 4 days following exposure to PLA2 enzymes, the duration of MV or weaning in severely affected patients may be prolonged (25, 26). Prompt antivenom administration is attributed to rapid neutralization of PLA2 enzymes resulting in improvement of clinical signs and may aid in the weaning process (45, 58).

Pressure support ventilation provides a set inspiratory pressure support to the patient's spontaneous breaths, which is gradually reduced until the patient demonstrates they can generate adequate tidal volumes with a low level of support (133). PSV has been shown in human medicine to have significantly fewer weaning failures compared to other methods (135, 136). The addition of automated tube compensation (ATC) to overcome endotracheal tube flow-resistance within PSV mode decreased time to weaning compared to PSV alone (137). ATC did not significantly affect other outcome measures, including occurrence of pneumonia, need for reintubation and mortality (137). PSV was successfully used in a single puppy with lower motor neuron disease but has not been specifically reported in snake envenomation (75).

In SIMV the patient is delivered a combination of spontaneous, supported breaths and mandatory breaths (133). Weaning is achieved by reducing the frequency of mandatory breaths delivered. There is evidence to suggest SIMV can worsen respiratory muscle fatigue and is reported to be a less successful method for weaning in the human literature (134, 138–140).

Tracheostomy tube placement may be considered in brachycephalic breeds or those with laryngeal paralysis, which is a possible consequence that has not yet been reported in snake envenomation (141). Tracheostomy placement can reduce the patient's sedation requirements which may be beneficial in the weaning period (142). A systematic review in humans found early tracheostomy tube placement in patients undergoing MV reduces sedative requirements but does not significantly affect incidence of VAP, duration of MV or mortality (143). A 1964 study of elapid envenomation described tracheostomy tube placement in 30% (16/52) of affected patients with common complications including minor tracheostomy site hemorrhage (11%, 6/52) tube obstruction and displacement (144), More recent literature does not report tracheostomy for snake envenomation, so the utility of this procedure in this context is unclear (61, 63, 64, 85, 87, 89, 129, 137, 144–147). Emergency cricothyroidotomy has been described in a person with crotalid envenomation when severe facial and oral oedema prevented successful orotracheal intubation (148). The patient was weaned from MV after 36 h without complication. Patients with temporary tracheostomies must remain closely monitored due to the risk of acute airway occlusion (69).

## 9. Outcomes

The overall prognosis for snake envenomated patients is good provided appropriate therapy is administered in the acute setting and there are minimal complications. Median survival to discharge for cats and dogs with elapid envenomation is 72% (76–84%) with 33 h (19.5–58 h) median duration of mechanical ventilation and 140 h (84–196 h) median hospitalization. To the authors' knowledge, no large-scale studies exist regarding envenomated patients receiving MV exist. Despite small population groups in the existing literature, patients requiring MV for elapid envenomation have a good prognosis, likely because they are primarily ventilated for hypoventilation due to a reversible underlying disease process.

The largest study of 42 dogs and cats treated in Australia reported a survival rate of 76% (32/42), which increased to 82% (32/39) with exclusion of cost-based euthanasia (58). Forty of these patients were ventilated for hypoventilation or unsustainable respiratory effort only, of which 84% (31/37) of patients survived following cost-based euthanasia exclusion (58). An Australian study on cats ventilated for elapid envenomation reported survival to discharge of 91.7% (11/12) (45). Seven dogs and one cat ventilated for *M. fulvius* envenomation had a survival rate of 87.5% (7/8) (19). Another study investigated 20 dogs and cats with coral snake envenomation, of which 4 dogs required MV (2). Two of these dogs were ventilated, with the survivor ventilated for 48 h, and the other dog euthanized after 24 h due to ARDS and AKI (2). A study of cats and dogs with *P. textilis* envenomation reported 25% (4/16) of affected patients required MV, of which 75% (3/4) of these patients survived to discharge, with the other euthanized due to unspecified hemorrhage (5). In a study of 91 cats and dogs with *P.porphyriacus* envenomation, 6.8% (6/88) dogs required mechanical ventilation, of which 66% (4/6) survived to discharge (4). Another dog was ventilated for 18 h following *P. porphyriacus* envenomation and made a full recovery (16). All studies described are retrospective and of a referral population. Survival in cats is much better than those of cats ventilated for other causes (45, 69, 149, 150). It is unclear why envenomated cats have a better prognosis, but it may be multifactorial including the reversibility of snake envenomation compared to other non-curable disease processes.

The reported duration of hospitalization of cats and dogs varied with medians of 84 and 196 h (45, 58). In the larger study, the patient population was ventilated primarily for *P. textilis* envenomation (24/42) and one each for *N. scutatis, A. antarcticus*, and *P. porphyriqcus* envenomation, however in 15/42 cats and dogs, the snake species was not identified (58). Duration of hospitalization for *M. fulvius* envenomation was 120 h (19).

Median duration of MV in survivors of Australian elapids has been reported as 19.5 and 20 h (45, 58). Another study reported median duration of MV in cats and dogs with *M. fulvius* envenomation as 58 h, however it is unclear if this was for survivors or all patients (19). The median time of MV in six dogs and cats with *P. porphyriacus* envenomation was 13.5 h, but included non-survivors (4). Another two dogs were ventilated for *P. porphyriacus* envenomation for 18 and 36 h respectively, but the latter was euthanized due to hemorrhage (8). One dog with *Oxyuranus* spp. envenomation was ventilated for 99 h before recovery, and two dogs with *Acanthophis* spp. envenomation were ventilated for 13 and 37 h before they were successfully weaned and discharged from hospital (88, 118). One dog with *Crotalus* spp. envenomation was ventilated for 76 h and was weaned from MV but suffered respiratory arrest after development of ARDS (123).

## 10. Areas of future research

The current veterinary literature examining mechanical ventilation in dogs and cats suffering from snake envenomation is limited to studies with small population sizes. Most studies focus on the management of envenomation as whole, rather than specifically on MV. Further research is warranted, particularly prospective studies addressing ventilator modes, weaning protocols and anesthetic practices. Investigation into the incidence of AKI would be of additional benefit given the potential for its development secondary to envenomation itself or as a complication of MV. Much of the available literature focuses on envenomation in dogs, with only one retrospective cat specific study identified. This study reported an excellent prognosis (45) in contrast to existing literature in ventilated cats (58, 69, 142, 149–151). Prospective studies investigating MV in snake envenomated cats are needed to validate these outcomes, which may improve owner willingness to pursue treatment.

## 11. Conclusion

Mechanical ventilation plays an important role in the management of patients suffering from the neurotoxic effects of snake envenomation. Despite available literature, no large cohort or prospective studies have been published at this time. Additional research in this area is needed.

From the limited data that is available, overall outcomes are favorable and severe complications are infrequent. Cats and dogs with snake envenomation that are successfully liberated from mechanical ventilation are likely to survive to discharge. Mechanical ventilation is an intensive care procedure and clinicians should discuss the risks, costs and prognosis with owners prior to commencement.

## Author contributions

CM and RD performed research, manuscript preparation, and editing. All authors contributed to the article and approved the submitted version.

## Footnotes

^1^Commonwealth Serum Laboratories Seqirus, Parkville, Victoria, Australia.

^2^Clinicare, Zoetis Inc, Kalamazoo, MI, USA.

^3^CliniCare RF, Zoetis Inc, Kalamazoo, MI, USA.

^4^Ensure, Abbott Australasia Pty Ltd., Macquarie Park, Australia.

^5^Convalescence Support Instant Diet, Royal Canin SAS, Gard, France.

^6^EnteralCare KC Canine, PetAg Inc., Hampshire, IL, USA.
